# Activity of Meropenem-Vaborbactam against Bacterial Isolates Causing Pneumonia in Patients in U.S. Hospitals during 2014 to 2018

**DOI:** 10.1128/AAC.02177-19

**Published:** 2020-02-21

**Authors:** Cecilia G. Carvalhaes, Dee Shortridge, Helio S. Sader, Mariana Castanheira

**Affiliations:** a JMI Laboratories, North Liberty, Iowa, USA

**Keywords:** CPE, CRE, *Enterobacterales*, *Enterobacteriaceae*, HAP, *Pseudomonas aeruginosa*, VAP, carbapenemase, hospital-acquired pneumonia, ventilator-acquired pneumonia

## Abstract

Meropenem-vaborbactam is approved to treat hospital-acquired pneumonia (HAP), including ventilator-associated pneumonia (VAP), in Europe. Meropenem-vaborbactam activity was evaluated against 3,193 Pseudomonas aeruginosa and 4,790 *Enterobacterales* isolates causing pneumonia, including VAP, in hospitalized patients in the United States. Susceptibility testing was performed by using the broth microdilution method, and all carbapenem-resistant isolates were submitted for whole-genome sequencing. Meropenem-vaborbactam exhibited almost complete activity against *Enterobacterales* (>99.9% susceptible), including carbapenem-resistant *Enterobacterales* (CRE), and was also very active against P. aeruginosa isolates (89.5% susceptible).

## Abstract (precis)

Meropenem-vaborbactam is approved to treat hospital-acquired pneumonia (HAP), including ventilator-associated pneumonia (VAP), in Europe. Meropenem-vaborbactam activity was evaluated against 3,193 Pseudomonas aeruginosa and 4,790 *Enterobacterales* isolates causing pneumonia, including VAP, in hospitalized patients in the United States. Susceptibility testing was performed by using the broth microdilution method, and all carbapenem-resistant isolates were submitted for whole-genome sequencing.

## INTRODUCTION

Hospital-acquired pneumonia (HAP) and ventilator associated pneumonia (VAP) represent major causes of mortality and resource utilization in hospitalized patients (1, 2). Although Staphylococcus aureus, Pseudomonas aeruginosa, and *Enterobacterales* remain important causes of pneumonia in hospitalized patients (PHP), their susceptibility patterns have varied markedly over time and among geographical regions, and choosing an empirical therapy based on whether the patient is at a high or low risk for multidrug-resistant (MDR) infections is challenging (3–5).

Meropenem-vaborbactam was recently approved by the European Medicines Agency (EMA) for the treatment of HAP, including VAP, in addition to the treatment of complicated intra-abdominal and urinary tract infections and acute pyelonephritis. Meropenem-vaborbactam was also approved for bacteremia that occurs in association with any of these infections and infections due to aerobic Gram-negative organisms where treatment options are limited (24). In the United States, meropenem-vaborbactam is approved for the treatment of complicated urinary tract infections, including pyelonephritis (7).

This study evaluated the *in vitro* activity of meropenem-vaborbactam against 4,790 *Enterobacterales* and 3,193 P. aeruginosa isolates causing pneumonia in hospitalized patients (PHP) from 31 U.S. hospitals distributed among 22 states from all 9 census divisions during 2014 to 2018.

Isolates were tested for susceptibility to meropenem-vaborbactam (inhibitor at a fixed concentration of 8 mg/liter) and comparator agents at JMI Laboratories (North Liberty, IA) by reference broth microdilution (6). Quality control and results interpretation were performed in accordance with CLSI, EUCAST (meropenem-vaborbactam against P. aeruginosa; colistin against *Enterobacterales*), or the U.S. FDA antibacterial susceptibility test interpretative criteria (tigecycline against *Enterobacterales*) (8–10).

Meropenem-vaborbactam was very potent against the entire collection of *Enterobacterales* (MIC_50/90_, 0.03/0.06 mg/liter) isolates and inhibited >99.9% (4,788/4,790) of those isolates. Amikacin (98.7%), carbapenems (meropenem, 97.2%; imipenem, 92.8%), and tigecycline (96.6%) (Table 1) also showed susceptibility rates of >90%. Ceftriaxone, levofloxacin, piperacillin-tazobactam, and cefepime inhibited 77.7%, 80.7%, 87.3%, and 87.8% of *Enterobacterales* isolates, respectively, when applying CLSI breakpoints. Meropenem-vaborbactam MIC_90_ values were 32- to 256-fold lower than the established susceptibility breakpoints (CLSI, ≤4/8 mg/liter; EUCAST, ≤8/8 mg/liter), regardless of the *Enterobacterales* species: K. pneumoniae (*n* = 1,219; MIC_90_, 0.03 mg/liter), Escherichia coli (*n* = 919; MIC_90_, 0.03 mg/liter), Serratia marcescens (*n* = 665, MIC_90_, 0.06 mg/liter), Enterobacter cloacae species complex (*n* = 649, MIC_90_, 0.03 mg/liter), Klebsiella aerogenes (*n* = 347, MIC_90_, 0.03/0.03 mg/liter), and Proteus mirabilis (*n* = 211, MIC_90_, 0.12 mg/liter).

**TABLE 1:** Antimicrobial susceptibility of *Enterobacterales*, P. aeruginosa and resistant subsets collected in 2014–2018 from patients hospitalized with pneumonia and VAP

Antimicrobial agent	PHP					VAP				
	MIC (mg/liter)		*N*	CLSI (%)^*a*^		MIC (mg/liter)		*N*	CLSI (%)^*a*^	
	50%	90%		S	R	50%	90%		S	R
*Enterobacterales*			4,790					814		
Meropenem-vaborbactam	0.03	0.06		>99.9	<0.1	0.03	0.06		100.0	0.0
Meropenem	0.03	0.06		97.2	2.3	0.03	0.06		98.3	1.5
Imipenem	0.25	1		92.8	3.6	0.25	1		94.3	2.2
Cefepime	≤0.5	8		87.8^*b*^	9.2	≤0.5	2		92.4	5.5
Ceftazidime	0.25	32		82.8	15.6	0.25	32		85.3	13.4
Ceftriaxone	0.12	>8		77.7	20.6	0.12	>8		80.6	17.2
Piperacillin-tazobactam	2	64		87.3	7.1	2	64		87.0	7.5
Aztreonam	≤0.12	>16		82.3	16.4	≤0.12	>16		84.0	14.6
Amikacin	2	4		98.7	0.3	2	4		99.1	0.1
Gentamicin	≤1	2		91.3	7.5	≤1	≤1		95.3	3.6
Tigecycline^*c*^	0.25	1		96.6	0.3	0.25	1		97.1	0.2
Levofloxacin	≤0.12	>4		80.7	16.8	≤0.12	>4		84.3	12.8
Colistin^*d*^	≤0.5	>8		76.1	23.9	≤0.5	>8		77.8	22.2
CRE^*e*^			131					13		
Meropenem-vaborbactam	0.03	0.5		98.5	0.8	0.06	1		100.0	0.0
Meropenem	16	>32		3.8	85.5	4	32		0.0	92.3
Imipenem	>8	>8		0.0	98.5	8	>8		0.0	84.6
Cefepime	>16	>16		8.4^*c*^	77.9	16	>16		30.8	53.8
Ceftazidime	>32	>32		4.6	93.1	>32	>32		15.4	76.9
Ceftriaxone	>8	>8		2.3	96.9	>8	>8		0.0	92.3
Piperacillin-tazobactam	>64	>64		3.8	89.3	>64	>64		7.7	61.5
Aztreonam	>16	>16		1.5	96.9	>16	>16		7.7	84.6
Amikacin	8	32		73.3	6.1	2	32		84.6	7.7
Gentamicin	4	>8		52.7	26.7	≤1	>8		76.9	15.4
Tigecycline^*c*^	0.5	2		96.9	1.5	0.5	1		100.0	0.0
Levofloxacin	>4	>4		16.8	79.4	0.5	>4		53.8	38.5
Colistin^*d*^	≤0.5	>8		76.9	23.1	≤0.5	>8		84.6	15.4
Pseudomonas aeruginosa			3,193					545		
Meropenem-vaborbactam^*d*^	0.5	16		89.5	10.5	0.5	16		88.8	11.2
Meropenem	0.5	16		76.4	16.9	0.5	16		73.8	10.3
Imipenem	1	>8		74.5	21.4	1	>8		77.2	22.8
Cefepime	4	16		82.4	6.1	4	16		82.5	5.1
Ceftazidime	2	32		81.7	13.2	2	32		82.4	12.7
Piperacillin-tazobactam	4	>64		77.5	11.7	8	>64		74.3	11.9
Aztreonam	8	>16		66.5	21.9	8	>16		63.7	23.5
Amikacin	4	16		94.2	3.3	4	8		96.9	1.3
Gentamicin	2	>8		82.5	10.3	2	8		85.0	9.0
Levofloxacin	1	>4		62.0	26.7	0.5	>4		67.7	23.9
Colistin	1	2		99.7	0.3	1	2		99.8	0.2
MDR^*f*^ P. aeruginosa			697					124		
Meropenem-vaborbactam^*d*^	8	32		59.0	41.0	8	32		59.7	40.3
Meropenem	8	32		22.1	63.1	8	32		21.0	38.7
Imipenem	8	>8		22.8	69.6	8	>8		23.4	70.2
Cefepime	16	>16		32.9	24.7	16	>16		34.7	20.2
Ceftazidime	16	>32		35.3	48.5	16	>32		46.8	41.9
Piperacillin-tazobactam	64	>64		23.0	43.6	64	>64		19.4	41.1
Aztreonam	>16	>16		16.8	66.7	>16	>16		15.3	67.7
Amikacin	8	>32		80.8	12.1	8	16		90.3	3.2
Gentamicin	8	>16		44.9	35.7	8	16		49.2	33.1
Levofloxacin	>4	>4		9.2	74.5	>4	>4		14.5	69.4
Colistin	0.5	2		99.1	0.9	1	2		100.0	0.0
XDR^*g*^ P. aeruginosa			440					70		
Meropenem-vaborbactam^*d*^	16	32		48.6	51.4	16	32		47.1	52.9
Meropenem	16	32		10.0	76.6	16	32		10.0	72.9
Imipenem	>8	>8		13.2	79.5	8	>8		15.7	77.1
Cefepime	16	>16		18.2	34.1	16	>16		20.0	24.3
Ceftazidime	32	>32		23.9	58.4	16	>32		44.3	44.3
Piperacillin-tazobactam	>64	>64		8.9	53.2	64	>64		2.9	48.6
Aztreonam	>16	>16		9.5	77.3	>16	>16		10.0	78.6
Amikacin	8	>32		76.8	23.2	8	32		87.1	5.7
Gentamicin	8	>16		37.0	41.8	8	>16		40.0	40.0
Levofloxacin	>4	>4		2.5	83.6	>4	>4		1.4	81.4
Colistin	0.5	1		99.1	0.9	1	2		100.0	0.0

a Criteria as published by CLSI (38).

b Intermediate interpreted as susceptible-dose dependent.

c FDA breakpoints (39).

*^d^* Criteria as published by EUCAST (37).

e CRE, carbapenem-resistant *Enterobacterales*.

f MDR, multidrug resistant.

g XDR, extensively drug resistant.

Carbapenem resistance was observed in a total of 131 (2.7%) PHP and 13 (1.6%) VAP *Enterobacterales* isolates, and these rates were similar to data published previously (11, 12). Among all antimicrobial agents tested, only meropenem-vaborbactam (MIC_50_/MIC_90_, 0.03/0.5 mg/liter; 98.5% susceptible) and tigecycline (MIC_50_/MIC_90_, 0.5/2 mg/liter; 96.9% susceptible) (Table 1) were active against >90% of carbapenem-resistant *Enterobacterales* (CRE) isolates. Colistin, amikacin, and gentamicin showed activity against 76.9%, 73.3%, and 52.7% of these isolates, respectively (Table 1). All other antimicrobials tested had limited activity against CRE isolates (<20%). All CRE isolates recovered from patients with VAP were susceptible to meropenem-vaborbactam (100%) (Table 1), and 84.6% displayed colistin and amikacin susceptible profiles. Levofloxacin was active against 53.8% of the CRE isolates causing VAP but had very limited activity against PHP isolates (16.8%) (Table 1).

Isolates that met the CRE criteria were submitted for whole-genome sequencing and analysis as previously described (13). Carbapenemase-encoding genes were detected in 53.4% (70/131) of CRE isolates, and this finding corroborates those from previous national studies (11, 12). Klebsiella pneumoniae carbapenemase (KPC; 94.2% [66/70]) remained the most frequent carbapenemase detected among carbapenemase-producing *Enterobacterales* (CPE) isolates causing PHP (Table 2). Unlike other carbapenemase enzymes that have been infrequently reported in U.S. hospitals, KPC-producing isolates have been reported in every U.S. state, though the endemicity of KPC-producing bacteria within the United States remains focused in regional hot spots (4, 12, 14). In this study, approximately two-thirds of the KPC-producing *Enterobacterales* isolates detected were from the Middle Atlantic region, although these isolates were also observed in most U.S. census divisions. Meropenem-vaborbactam (MIC_50/90_, 0.03/0.5 mg/liter) was 512-fold more active than meropenem (MIC_50/90_, 16/>32 mg/liter) against KPC-producing isolates based on MIC_50_ values. These findings are in agreement with previous results where the combination of vaborbactam reduces meropenem MIC values >64-fold for CPE isolates (15–17).

**TABLE 2:** Distribution of carbapenemase genes detected among *Enterobacterales* isolates causing pneumonia in hospitalized patients in U.S. medical centers (2014 to 2018)

Organism	Carbapenemase detected	No. of isolates	US census division(s)	MIC range (mg/liter)	
				Meropenem	Meropenem-vaborbactam
Citrobacter freundii species complex	KPC-2	1	Middle Atlantic	2	<0.015
	KPC-3	2	Middle Atlantic	8 to 16	0.03
Klebsiella oxytoca	KPC-2	2	Mountain, East North Central	2 to 32	0.03
	KPC-3	4	Middle Atlantic, South Atlantic	1 to 32	0.03
Klebsiella pneumoniae	KPC-2	21	Middle Atlantic, West South Central	1 to >32	<0.015 to 2
	KPC-3	19	Middle Atlantic, East North Central, Pacific	2 to >32	<0.015 to 1
Enterobacter cloacae species complex	KPC-3	10	New England, Middle Atlantic, Mountain	2 to >32	0.03 to 0.25
					
Escherichia coli	KPC-3	1	Middle Atlantic	16	0.03
Serratia marcescens	KPC-3	6	Middle Atlantic, East North Central	2 to >32	0.06 to 1
	SME-4	2	Mountain, Middle Atlantic	>32	0.03 to 0.06
	NDM-1	1	Middle Atlantic	4	8
Proteus mirabilis	IMP-64	1	Mountain	16	16

All KPC-producing isolates were inhibited by meropenem-vaborbactam regardless of the KPC variant produced. KPC-3 (*n* = 42; 60.9% of all CPE) was more common than KPC-2 (*n* = 24; 34.8%) and was disseminated among 6 *Enterobacterales* species from all U.S. census divisions except West North Central, East South Central, and West South Central (Table 2). In contrast, KPC-2 was detected mainly in K. pneumoniae isolates and from 4 U.S. census divisions: Middle Atlantic (17 isolates), West South Central (5 isolates), East North Central (1 isolate), and Mountain (1 isolate). Of note, meropenem-vaborbactam showed similar activity against K. pneumoniae isolates carrying KPC-3 (MIC_50_/MIC_90,_ 0.03/0.5 mg/liter) or KPC-2 (MIC_50_/MIC_90,_ 0.03/1 mg/liter), in contrast to data published by Satlin and colleagues that showed higher ceftazidime-avibactam MIC values against KPC-3 producers (18).

Meropenem-vaborbactam (97.1% susceptible) displayed activity against all CRE isolates except 1 NDM-1-producing S. marcescens (MIC, 8 mg/liter) from Middle Atlantic and 1 IMP-64-producing P. mirabilis (MIC, 16 mg/liter) (Table 1) from Mountain divisions. Vaborbactam is a potent inhibitor of serine β-lactamases, but the agent lacks activity against metallo-β-lactamases (MBLs) and class D carbapenemase (19). In addition to KPC enzymes, SME-4-encoding genes (*n* = 2) were also detected in S. marcescens isolates from Middle Atlantic and Mountain divisions, and meropenem-vaborbactam inhibited both isolates at an MIC of ≤0.06 mg/liter (Table 2). No class D carbapenemase genes were detected among CRE isolates.

No carbapenemase genes were observed in 61 CRE isolates (46.6%), and meropenem-vaborbactam was the only agent tested to inhibit 100% of these isolates. Tigecycline, colistin, and amikacin were active against 98.4%, 75.4%, and 68.9%, respectively. Limited activity was observed for all β-lactams agents, including meropenem (MIC_50/90_, 8/>32 mg/liter; 4.9% susceptible). Resistance mechanisms other than carbapenemase production, such as lack of major porins and overexpression of AcrAB-TolC efflux pumps combined with extended spectrum cephalosporinases or AmpC production, are well known causes of meropenem resistance. Some of those, in addition to an increase in the *bla*_KPC_ gene copy number, were described to possibly affect meropenem-vaborbactam activity (19, 20). However, these mechanisms can be overcome by targeting *in vivo* exposures that maximize the efficacy of the meropenem-vaborbactam combination. Recently completed clinical trials demonstrated that these target exposures appear to be achievable due to the excellent safety profiles of both meropenem and vaborbactam (21–23).

Results of the phase 3 clinical trial (Tango II) to evaluate the safety, efficacy, and tolerability of meropenem-vaborbactam monotherapy in treating patients with serious CRE infections versus best available therapy (BAT) were very encouraging (23). Patients randomized to the meropenem-vaborbactam arm received 7 to 14 days of treatment as monotherapy (2 g-2 g) via intravenous infusion over 3 h every 8 h, and BAT therapy included polymyxins, carbapenems, aminoglycosides, or tigecycline as monotherapy or in combination and ceftazidime-avibactam monotherapy. Day 28 all-cause mortality was 15.6% (5/32) and 33.3% (5/15) for meropenem-vaborbactam and BAT, respectively. Although only 5 patients with HAP/VAP were included, meropenem-vaborbactam is a promising β-lactam/β-lactam-inhibitor combination for treating pathogens causing HAP and VAP, including CRE infections, and this combination compound gained EMA approval for these indications (24).

The findings of this study, where meropenem-vaborbactam, aminoglycosides, carbapenems, and tigecycline were the only agents displaying susceptibility rates >90% against 4,790 *Enterobacterales* isolates, reinforce the challenges to improve care for patients with HAP/VAP, for which delayed and inadequate treatments have been associated with increased rates of morbidity and mortality (25, 26). Similar results were observed when these agents were tested against a worldwide collection of *Enterobacterales* recovered from different infection sources (12). The emergence and widespread geography of CRE isolates have added considerable challenges to treating severe infections, and mortality rates are as high as 40% to 50% (27–29). Therapeutic options to treat CRE HAP/VAP infections are limited, and traditionally, agents from either the polymyxin or aminoglycoside classes have been recommended in combined therapy, usually with carbapenem-containing regimens (1, 26, 30, 31). However, studies have shown that colistin, tigecycline, and gentamicin have poor lung penetration, whereas carbapenems have good distribution in lungs, achieving clinically relevant concentrations (26, 32). In fact, herein, only meropenem-vaborbactam (98.5%) and tigecycline (96.9%) displayed >90% susceptibility rates against CRE isolates causing PHP.

P. aeruginosa isolates were recovered from 3,193 PHP, including 545 isolates deemed to cause VAP. Overall, 89.5% of P. aeruginosa isolates were inhibited at the meropenem-vaborbactam susceptible breakpoint established by EUCAST (≤8 mg/liter) compared to 76.4% susceptible to meropenem alone (at ≤2 mg/liter) (Tables 1 and 2). Colistin (MIC_50/90_, 1/2 mg/liter; 99.7% susceptible), amikacin (MIC_50/90_, 4/16 mg/liter; 94.2% susceptible), and meropenem-vaborbactam (MIC_50/90_, 0.5/16 mg/liter) were the most active agents against those isolates, followed by gentamicin (MIC_50/90_, 2/>8 mg/liter; 82.5% susceptible) and cephalosporins (cefepime: MIC_50/90_, 4/16 mg/liter; 82.4% susceptible; and ceftazidime: MIC_50/90_, 2/32 mg/liter; 81.7% susceptible). MDR and extensively drug-resistant (XDR) phenotypes (33, 34) were observed among 697 (21.8%) and 440 (13.8%) respective P. aeruginosa isolates, and meropenem-vaborbactam was the most active β-lactam agent tested, inhibiting 59.0% and 48.6% of these highly resistant pathogens, respectively (Table 1). Colistin was the only compound active against >90% of MDR (MIC_50/90_, 0.5/2 mg/liter; 99.1% susceptible) and XDR (MIC_50/90_, 0.5/1 mg/liter; 99.1% susceptible) subsets, followed by amikacin (MIC_50/90_, 8/>32 mg/liter; 80.8 to 76.8% susceptible). However, colistin and aminoglycoside therapy raise concerns on ensuring that therapeutic and nontoxic levels will be delivered to the patient (23). Similar susceptibility rates were observed between P. aeruginosa isolates recovered from patients with PHP and VAP (Table 1).

Facing the epidemic of multidrug-resistant Gram-negative bacilli, carbapenems have become the most empirically prescribed β-lactams in intensive care units for HAP/VAP in many geographic regions (35, 36). However, the meropenem standard dosage (1 g every 8 h, 30-min infusion) used to treat P. aeruginosa infections showed lower coverage (76.4% susceptible) against these isolates than the coverage observed by meropenem-vaborbactam (89.5% susceptible) when the approved dosage (2 g-2 g via intravenous [i.v.] infusion over 3 h every 8 h) and current EUCAST breakpoints were applied (37).

In summary, meropenem-vaborbactam was very active against a large collection of *Enterobacterales* isolates recovered from PHP and VAP in 31 U.S. hospitals over a 4-year period. This collection included CRE isolates that were resistant to many comparator agents but mostly (>99%) susceptible to meropenem-vaborbactam. Meropenem-vaborbactam was also active against P. aeruginosa isolates that were resistant to many antipseudomonal agents and had high MDR and XDR rates. This combination agent may be considered an effective alternative for the treatment of HAP/VAP infections in U.S. hospitals when the FDA approves that indication.

## References

[1] Kalil AC, Metersky ML, Klompas M, Muscedere J, Sweeney DA, Palmer LB, Napolitano LM, O'Grady NP, Bartlett JG, Carratalà J, El Solh AA, Ewig S, Fey PD, File TM, Restrepo MI, Roberts JA, Waterer GW, Cruse P, Knight SL, Brozek JL 2016 Management of adults with hospital-acquired and ventilator-associated pneumonia: 2016 clinical practice guidelines by the Infectious Diseases Society of America and the American Thoracic Society. Clin Infect Dis 63:e61–e111. doi:10.1093/cid/ciw353. 27418577 PMC4981759

[2] Kollef MH, Hamilton CW, Ernst FR 2012 Economic impact of ventilator-associated pneumonia in a large matched cohort. Infect Control Hosp Epidemiol 33:250–256. doi:10.1086/664049. 22314062

[3] Sader HS, Castanheira M, Arends SJR, Goossens H, Flamm RK 2019 Geographical and temporal variation in the frequency and antimicrobial susceptibility of bacteria isolated from patients hospitalized with bacterial pneumonia: results from 20 years of the SENTRY Antimicrobial Surveillance Program (1997–2016). J Antimicrob Chemother 74:1595–1606. doi:10.1093/jac/dkz074. 30843070

[4] Weiner LM, Webb AK, Limbago B, Dudeck MA, Patel J, Kallen AJ, Edwards JR, Sievert DM 2016 Antimicrobial-resistant pathogens associated with healthcare-associated infections: summary of data reported to the National Healthcare Safety Network at the Centers for Disease Control and Prevention, 2011-2014. Infect Control Hosp Epidemiol 37:1288–1301. doi:10.1017/ice.2016.174. 27573805 PMC6857725

[5] Tangden T, Giske CG 2015 Global dissemination of extensively drug-resistant carbapenemase-producing *Enterobacteriaceae*: clinical perspectives on detection, treatment and infection control. J Intern Med 277:501–512. doi:10.1111/joim.12342. 25556628

[6] CLSI. 2018 M07Ed11. Methods for dilution antimicrobial susceptibility tests for bacteria that grow aerobically; approved standard, 11th ed Clinical and Laboratory Standards Institute, Wayne, PA.

[7] FDA. 2017 VABOMERE (meropenem/vaborbactam) for injection prescribing information. https://www.accessdata.fda.gov/drugsatfda_docs/label/2017/209776lbl.pdf.

[8] CLSI. 2018 M100Ed28. Performance standards for antimicrobial susceptibility testing: 28th informational supplement. Clinical and Laboratory Standards Institute, Wayne, PA.

[9] EUCAST. 2018 Breakpoint tables for interpretation of MIC’s and zone diameters. Version 8.1, May 2018. http://www.eucast.org/fileadmin/src/media/PDFs/EUCAST_files/Breakpoint_tables/v_8.0_Breakpoint_Tables.pdf.

[10] USFDA. 2019 Antibacterial susceptibility test interpretive criteria. https://www.fda.gov/drugs/development-resources/antibacterial-susceptibility-test-interpretive-criteria. Accessed 5 May 2019.

[11] Sader HS, Castanheira M, Mendes RE, Flamm RK 2018 Frequency and antimicrobial susceptibility of Gram-negative bacteria isolated from patients with pneumonia hospitalized in ICUs of US medical centres (2015-17). J Antimicrob Chemother 73:3053–3059. doi:10.1093/jac/dky279. 30060117

[12] Castanheira M, Huband MD, Mendes RE, Flamm RK 2017 Meropenem-vaborbactam tested against contemporary Gram-negative isolates collected worldwide during 2014, including carbapenem-resistant, KPC-producing, multidrug-resistant, and extensively drug-resistant *Enterobacteriaceae*. Antimicrob Agents Chemother 61:e00567-17. doi:10.1128/AAC.00567-17. 28652234 PMC5571304

[13] Castanheira M, Doyle TB, Kantro V, Mendes RE, Shortridge D 11 11 2019 Meropenem-vaborbactam activity against carbapenem-resistant *Enterobacterales* isolates collected in U.S. hospitals during 2016–2018. Antimicrob Agents Chemother. doi:10.1128/AAC.01951-19. PMC6985738 31712207

[14] CDC. 2017 Tracking CRE. https://www.cdc.gov/hai/organisms/cre/trackingcre.html. Accessed 15 September 2019.

[15] Hecker SJ, Reddy KR, Totrov M, Hirst GC, Lomovskaya O, Griffith DC, King P, Tsivkovski R, Sun D, Sabet M, Tarazi Z, Clifton MC, Atkins K, Raymond A, Potts KT, Abendroth J, Boyer SH, Loutit JS, Morgan EE, Durso S, Dudley MN 2015 Discovery of a cyclic boronic acid beta-lactamase inhibitor (RPX7009) with utility vs class A serine carbapenemases. J Med Chem 58:3682–3692. doi:10.1021/acs.jmedchem.5b00127. 25782055

[16] Castanheira M, Rhomberg PR, Flamm RK, Jones RN 2016 Effect of the beta-lactamase inhibitor vaborbactam combined with meropenem against serine carbapenemase-producing *Enterobacteriaceae*. Antimicrob Agents Chemother 60:5454–5458. doi:10.1128/AAC.00711-16. 27381386 PMC4997822

[17] Hackel MA, Lomovskaya O, Dudley MN, Karlowsky JA, Sahm DF 2017 *In vitro* activity of meropenem-vaborbactam against clinical isolates of KPC-positive *Enterobacteriaceae*. Antimicrob Agents Chemother 62:e01904-17. doi:10.1128/AAC.01904-17. 29084745 PMC5740317

[18] Satlin MJ, Chen L, Patel G, Gomez-Simmonds A, Weston G, Kim AC, Seo SK, Rosenthal ME, Sperber SJ, Jenkins SG, Hamula CL, Uhlemann AC, Levi MH, Fries BC, Tang YW, Juretschko S, Rojtman AD, Hong T, Mathema B, Jacobs MR, Walsh TJ, Bonomo RA, Kreiswirth BN 2017 Multicenter clinical and molecular epidemiological analysis of bacteremia due to carbapenem-resistant *Enterobacteriaceae* (CRE) in the CRE epicenter of the United States. Antimicrob Agents Chemother 61:e02349-16. doi:10.1128/AAC.02349-16. 28167547 PMC5365653

[19] Lomovskaya O, Sun D, Rubio-Aparicio D, Nelson K, Tsivkovski R, Griffith DC, Dudley MN 2017 Vaborbactam: spectrum of beta-lactamase inhibition and impact of resistance mechanisms on activity in *Enterobacteriaceae*. Antimicrob Agents Chemother 61:e01443-17. doi:10.1128/AAC.01443-17. 28848018 PMC5655098

[20] Sun D, Rubio-Aparicio D, Nelson K, Dudley MN, Lomovskaya O 2017 Meropenem-vaborbactam resistance selection, resistance prevention, and molecular mechanisms in mutants of KPC-producing *Klebsiella pneumoniae*. Antimicrob Agents Chemother 61:e01694-17. doi:10.1128/AAC.01694-17. 29038260 PMC5700310

[21] Griffith DC, Loutit JS, Morgan EE, Durso S, Dudley MN 2016 Phase 1 study of the safety, tolerability, and pharmacokinetics of the beta-lactamase inhibitor vaborbactam (RPX7009) in healthy adult subjects. Antimicrob Agents Chemother 60:6326–6332. doi:10.1128/AAC.00568-16. 27527080 PMC5038296

[22] Bhavnani SM, Hammel JP, Rubino CM, Trang M, Loutit JS, Griffith DC, Lomovskaya O, Dudley MN, Ambrose PG 2017 Meropenem-vaborbactam pharmacokinetic-pharmacodymanic analyses for efficacy based on data from patients enrolled in phase 3 studies, poster Sunday-193. 2nd ASM Microbe, 1 to 5 June, New Orleans, LA.

[23] Wunderink RG, Giamarellos-Bourboulis EJ, Rahav G, Mathers AJ, Bassetti M, Vazquez J, Cornely OA, Solomkin J, Bhowmick T, Bishara J, Daikos GL, Felton T, Furst MJL, Kwak EJ, Menichetti F, Oren I, Alexander EL, Griffith D, Lomovskaya O, Loutit J, Zhang S, Dudley MN, Kaye KS 2018 Effect and safety of meropenem-vaborbactam versus best-available therapy in patients with carbapenem-resistant *Enterobacteriaceae* infections: the TANGO II randomized clinical trial. Infect Dis Ther 7:439–455. doi:10.1007/s40121-018-0214-1. 30270406 PMC6249182

[24] EMA. 2018 Vaborem product information. https://www.ema.europa.eu/en/documents/product-information/vaborem-epar-product-information_en.pdf.

[25] Bassetti M, Welte T, Wunderink RG 2016 Treatment of Gram-negative pneumonia in the critical care setting: is the beta-lactam antibiotic backbone broken beyond repair? Crit Care 20:19. doi:10.1186/s13054-016-1197-5. 26821535 PMC4731981

[26] Bassetti M, Peghin M, Carnelutti A, Righi E 2017 How should we treat HAP/VAP caused by carbapenemase-producing *Enterobacteriaceae*? Semin Respir Crit Care Med 38:301–310. doi:10.1055/s-0037-1602656. 28578554

[27] Tumbarello M, Viale P, Viscoli C, Trecarichi EM, Tumietto F, Marchese A, Spanu T, Ambretti S, Ginocchio F, Cristini F, Losito AR, Tedeschi S, Cauda R, Bassetti M 2012 Predictors of mortality in bloodstream infections caused by *Klebsiella pneumoniae* carbapenemase-producing *K. pneumoniae*: importance of combination therapy. Clin Infect Dis 55:943–950. doi:10.1093/cid/cis588. 22752516

[28] CDC. 2013 Vital signs: carbapenem-resistant *Enterobacteriaceae*. MMWR Morb Mortal Wkly Rep 62:165–170. 23466435 PMC4604788

[29] CDC. 2013 Antibiotic resistance threats in the United States, 2013. http://www.cdc.gov/drugresistance/pdf/ar-threats-2013-508.pdf.

[30] Garnacho-Montero J, Corcia-Palomo Y, Amaya-Villar R, Martin-Villen L 2014 How to treat VAP due to MDR pathogens in ICU patients. BMC Infect Dis 14:135. doi:10.1186/1471-2334-14-135. 25430700 PMC4289192

[31] Daikos GL, Tsaousi S, Tzouvelekis LS, Anyfantis I, Psichogiou M, Argyropoulou A, Stefanou I, Sypsa V, Miriagou V, Nepka M, Georgiadou S, Markogiannakis A, Goukos D, Skoutelis A 2014 Carbapenemase-producing *Klebsiella pneumoniae* bloodstream infections: lowering mortality by antibiotic combination schemes and the role of carbapenems. Antimicrob Agents Chemother 58:2322–2328. doi:10.1128/AAC.02166-13. 24514083 PMC4023796

[32] Doi Y, Paterson DL 2015 Carbapenemase-producing *Enterobacteriaceae*. Semin Respir Crit Care Med 36:74–84. doi:10.1055/s-0035-1544208. 25643272 PMC4470611

[33] Magiorakos AP, Srinivasan A, Carey RB, Carmeli Y, Falagas ME, Giske CG, Harbarth S, Hindler JF, Kahlmeter G, Olsson-Liljequist B, Paterson DL, Rice LB, Stelling J, Struelens MJ, Vatopoulos A, Weber JT, Monnet DL 2012 Multidrug-resistant, extensively drug-resistant and pandrug-resistant bacteria: an international expert proposal for interim standard definitions for acquired resistance. Clin Microbiol Infect 18:268–281. doi:10.1111/j.1469-0691.2011.03570.x. 21793988

[34] Farrell DJ, Flamm RK, Sader HS, Jones RN 2013 Antimicrobial activity of ceftolozane-tazobactam tested against *Enterobacteriaceae* and *Pseudomonas aeruginosa* with various resistance patterns isolated in U.S. Hospitals (2011–2012). Antimicrob Agents Chemother 57:6305–6310. doi:10.1128/AAC.01802-13. 24100499 PMC3837887

[35] Barbier F, Andremont A, Wolff M, Bouadma L 2013 Hospital-acquired pneumonia and ventilator-associated pneumonia: recent advances in epidemiology and management. Curr Opin Pulm Med 19:216–228. doi:10.1097/MCP.0b013e32835f27be. 23524477

[36] Rello J, Ulldemolins M, Lisboa T, Koulenti D, Manez R, Martin-Loeches I, De Waele JJ, Putensen C, Guven M, Deja M, Diaz E, EU-VAP/CAP Study Group. 2011 Determinants of prescription and choice of empirical therapy for hospital-acquired and ventilator-associated pneumonia. Eur Respir J 37:1332–1339. doi:10.1183/09031936.00093010. 20847075

[37] EUCAST. 2019 Breakpoint tables for interpretation of MICs and zone diameters. Version 9.0, January 2019. http://www.eucast.org/fileadmin/src/media/PDFs/EUCAST_files/Breakpoint_tables/v_9.0_Breakpoint_Tables.pdf.

[38] CLSI. 2019 M100Ed29. Performance standards for antimicrobial susceptibility testing: 29th informational supplement. Clinical and Laboratory Standards Institute, Wayne, PA.

[39] 2018 Tygacil product information. https://www.accessdata.fda.gov/drugsatfda_docs/label/2018/211158s000lbl.pdf.

